# Supplementary material for: Effectiveness of Postnatal Maternal or Caregiver Interventions on Outcomes among Infants under Six Months with Growth Faltering: A Systematic Review
Source: Nutrients. 2024 Mar 14;16(6):837. doi: 10.3390/nu16060837 (PMC10974267; doi:10.3390/nu16060837)
Supplement: Supplementary file 1 [file nutrients-16-00837-s001.zip › S1 File Search Strategy_FinalProofread-author_13032024.pdf]

Search strategy

Broad search terms

Concept 1: Population

Mothers of infants under 6 months of age: *maternal, mother*, lactat*, parent*, famil*, postnatal, carer, caregiver, father, grandmother, grandparents, wasting, wasted, WLZ, WHZ, underweight, WAZ, low mid upper arm circumference, MUAC, preterm, <37 weeks gestation, small size at birth, small for gestational age at birth, low birth weight, losing weight*

Concept 2: Intervention

KMC: *kangaroo mother care, KMC, Kangaroo care, skin to skin care, Skin-to-Skin Contact (SSC)*

Breastfeeding counselling and education intervention: *counselling, education, nutrition education, health education, breastfeeding, complementary feeding, nutrition counselling, postnatal breastfeeding education, postnatal care, maternal education, caregiver education, educational status, nutritional status*

Maternal nutritional supplementation: *supplement, folate, folic acid, iron, iron folic acid, IFA, calcium, iodine, vitamin D, zinc multiple micronutrients, multiple micro-nutrients, multi nutrient, multi-vitamin, multi-mineral, lipid-based nutrient supplement, LNS, balanced energy protein, macronutrient, poly-unsaturated fatty-acids, PUFA, long chain poly-unsaturated fatty-acids, LCPUFA, n-3 LCPUFA, n-3 PUFA, biofortified, biofortification, maternal nutrition supplements, dietary supplements, iodides, docosahexaenoic acid (dha), choline, omega-3 fatty acids*

Mental health: *psychosocial, psycho-social, maternal mental health, maternal depression, postpartum depression, peer support, social support, post traumatic stress disorder, ptsd, postpartum anxiety, postpartum psychological distress, puerperal depression, postnatal debriefing, psychoeducation, psychotherapy*

Relaxation therapy: *relaxation therapy, relaxation technique, meditation, music therapy, verbal protocol, guided imagery, mind stress-releasing therapy, relaxation lighting, imagery psychotherapy*

Cash transfer: *social safety net, cash transfers, child support grant, social transfer, social assistance, Maternity Benefit Program, Direct Benefit Transfers, public policy*

Women empowerment: *decision making, autonomy, empowerment, household decision making, women time use, social independence, Labour force participation, Mobility agency, Reproductive decisions*

Total articles retrieved from databases and trial registers search

SN	Name of the database	Last search date	Search results
1	APA PsycInfo (ovid)	02-08-2021	163
2	CINAHL Plus (EBSCO)	03-08-2021	982
3	Cochrane Library	02-08-2021	2839
4	Embase (ovid)	02-08-2021	2165

SN	Name of the database	Last search date	Search results
5	Global Health (ovid)	03-08-2021	798
6	Medline (PubMed)	04-08-2021	3225
7	Science direct	03-08-2021	3705
8	Scopus	03-08-2021	924
9	Web of science	04-08-2021	166
Total search results of databases			14,967
SN	Other information sources	Last search Date	Search results
1	BIOSIS previews	13-08-2021	645
2	ISRCTN	13-08-2021	74
3	WHO ICTRP	16-08-2021	1122
4	Clinicaltrials.gov	14-08-2021	300
Total search results of other information sources			2,141
Total Search Results= 17,108			

Detail Search Strategy for specific databases and other information sources

Database 1: APA PsycInfo

SN	Terms	Hits #
	Filters used: Canadian Agency for Drugs and Technology in Health [Internet]. Strings attached: CADTH's database search filters-randomized controlled trials / controlled clinical trials — OVID Medline, Embase, PsycINFO. Ottawa: CADTH; 2018. [cited 2020 Dec 09]. Available from: https://www.cadth.ca/resources/finding-evidence/strings-attached-cadths-database-search-filters#rand, Year: 2000 to 2021	
	Population terms	
1	((maternal or mother* or father or postnatal or lactat* or caregiver or grandmother) and (wasting or wasted or WLZ or WHZ or underweight or WAZ or "low mid upper arm circumference" or MUAC or preterm or "small size at birth" or "small for gestational age at birth" or "less than 37 weeks gestation" or "low birth weight" or "losing weight" or "infant near/5 six months" or "infant near/5 6 month")).mp. [mp=title, abstract, heading word, table of contents, key concepts, original title, tests & measures, mesh]	4695
2	limit 1 to yr="2000-2021"	3958
	Intervention terms-1 (Maternal Nutrition supplementation related)	
3	(folic acid or folate or iron or "iron folic acid" or "IFA").mp.	6254
4	(calcium or iodine or "vitamin D" or zinc).mp. [mp=title, abstract, heading word, table of contents, key concepts, original title, tests & measures, mesh]	22931
5	(m#cronutrient or vitamin or mineral).mp. [mp=title, abstract, heading word, table of contents, key concepts, original title, tests & measures, mesh]	9341
6	balanced energy protein.mp. [mp=title, abstract, heading word, table of contents, key concepts, original title, tests & measures, mesh]	0
7	("Polyunsaturated fatty acid" or PUFA or LCPUFA).mp. [mp=title, abstract, heading word, table of contents, key concepts, original title, tests & measures, mesh]	694

SN	Terms	Hits #
8	(Biofortifi* or iodides or Ergocalciferols or "docosahexaenoic acid" or DHA or choline or "omega-3 fatty acid").mp. [mp=title, abstract, heading word, table of contents, key concepts, original title, tests & measures, mesh]	4808
9	3 or 4 or 5 or 6 or 7 or 8	39103
	Intervention terms-1 (Combined with OR Boolean operator)	
10	2 and 9	122
	Intervention terms-2 (Education intervention related)	
11	((education or counselling or training or promotion) adj20 breastfeeding).mp. [mp=title, abstract, heading word, table of contents, key concepts, original title, tests & measures, mesh]	793
12	((education or counselling or training or promotion) adj20 complementary feeding).mp. [mp=title, abstract, heading word, table of contents, key concepts, original title, tests & measures, mesh]	14
13	((education or counselling or training or promotion) adj20 postnatal*).mp. [mp=title, abstract, heading word, table of contents, key concepts, original title, tests & measures, mesh]	412
14	11 or 12 or 13	1182
	Intervention terms-2 (Combined with OR Boolean operator)	
15	2 and 14	39
	Intervention terms-3 (Relaxation therapy related)	
16	(relaxation therapy or relaxation technique or meditation or music therapy or verbal protocol or guided imagery).mp. [mp=title, abstract, heading word, table of contents, key concepts, original title, tests & measures, mesh]	21730
	Intervention terms-3 (Combined with OR Boolean operator)	
17	2 and 16	29
	Intervention terms-4 (Mental Health interventions related)	
18	((psychosocial adj50 maternal) or (maternal mental health adj50 postnatal) or maternal depression or postpartum depression or peer support or peer-support or social support or Post-traumatic stress disorder or PTSD or Postpartum anxiety or Postpartum psychological distress or Puerperal depression or Baby blues or Postnatal Debriefing or postnatal Psychoeducation or postnatal Psychotherapy).mp. [mp=title, abstract, heading word, table of contents, key concepts, original title, tests & measures, mesh]	137966
	Intervention terms-4 (Combined with OR Boolean operator)	
19	2 and 18	499
	Intervention terms-5 (KMC related)	
20	("kangaroo mother care" or KMC or "kangaroo care" or "skin to skin care" or "Skin-to-Skin Contact").mp. [mp=title, abstract, heading word, table of contents, key concepts, original title, tests & measures, mesh]	359
	Intervention terms-5 (Combined with OR Boolean operator)	
21	2 and 20	103
	Intervention terms-6 (Women empowerment related)	
22	(empowerment or autonomy or household decision making or women time).mp. [mp=title, abstract, heading word, table of contents, key concepts, original title, tests & measures, mesh]	52557
	Intervention terms-6 (Combined with OR Boolean operator)	
23	2 and 22	40

SN	Terms	Hits #
	Intervention terms-7 (Cash Transfer related)	
24	(Social safety net or cash transfers or child support grant or Social transfer or social assistance or Maternity Benefit Programme or Direct Benefit Transfers, public policy).mp. [mp=title, abstract, heading word, table of contents, key concepts, original title, tests & measures, mesh]	894
	Intervention terms-7 (Combined with OR Boolean operator)	
25	2 and 24	4
	Population and Intervention terms	
26	10 or 15 or 17 or 19 or 21 or 23 or 25	792
27	exp Clinical Trials/ or Placebo/ or (random* or sham or placebo* or ((singl* or doubl*) adj (blind* or dumm* or mask*)) or ((tripl* or trebl*) adj (blind* or dumm* or mask*)) or (control* adj3 (study or studies or trial* or group*)) or Nonrandom* or non random* or non-random* or quasi-random* or quasirandom* or allocated or ((open label or open-label) adj5 (study or studies or trial*)) or ((equivalence or superiority or non-inferiority or noninferiority) adj3 (study or studies or trial*)) or ((pragmatic or practical) adj3 trial*) or ((quasiexperimental or quasi-experimental) adj3 (study or studies or trial*)) or (phase adj3 (III or "3") adj3 (study or studies or trial*))).ti,ab,hw.	368060
28	limit 26 to yr="2000-2021"	792
29	27 and 28	163

Database 2: CINAHL Plus

S	Terms	Hits #
	Filters used: Cochrane CINAHL Plus filter https://training.cochrane.org/handbook/current/chapter-04-technical-supplement-searching-and-selecting-studies#_Ref19195422 Published Date: 20000101-20211231	
S1	(MH (randomized controlled trials OR double-blind studies OR single-blind studies OR random assignment OR pretest-posttest design OR cluster sample) OR TI (randomised OR randomized) OR AB random* OR TI trial OR ((MH (sample size) AND AB (assigned OR allocated OR control))) OR MH (placebos OR crossover design OR comparative studies) OR AB ((control W5 group) OR (cluster W3 RCT) OR PT (randomized controlled trial))) NOT ((MH animals+ OR MH (animal studies) OR TI (animal model*)) NOT MH (human))	842477
	Population terms	
S2	TI (maternal or mother* or father or postnatal or lactat* or caregiver or carer or grandmother or parent) or AB (maternal or mother* or father or postnatal or lactat* or caregiver or carer or grandmother or parent)	330226
S3	TI (wasting or wasted or WLZ or WHZ or underweight or WAZ or "low mid upper arm circumference" or MUAC or preterm or "small size at birth" or "small for gestational age at birth" or "less than 37 weeks gestation" or "low birth weight" or "losing weight" or "infant near/5 six months" or "infant near/5 6 month") or AB (wasting or wasted or WLZ or WHZ or underweight or WAZ or "low mid upper arm circumference" or MUAC or preterm or "small size at birth" or "small for gestational age at birth" or "less than 37 weeks gestation" or	51348

S	Terms	Hits #
	"low birth weight" or "losing weight" or "infant near/5 six months" or "infant near/5 6 month")	
S4	(S2 AND S3) NOT (antenatal or "ante-natal")	15599
	Intervention terms-1 (Maternal Nutrition supplementation related)	
S5	supplement* or folic acid or folate or iron or "iron folic acid" or "IFA" or calcium or iodine or "vitamin D" or zinc or micronutrient or "multiple micronutrient" or vitamin or mineral or "balanced energy protein" or "Polyunsaturated fatty acid" or PUFA or LCPUFA or Biofortifi* or iodides or "docosahexaenoic acid" or DHA or "omega-3 fatty acid"	240588
	Intervention terms-2 (Education intervention related)	
S6	(counselling or education or "nutrition education" or "health education" or promotion or training) N30 ("breastfeeding" or "breast feeding" or "breast-feeding" or "complementary feeding" or postnatal or feeding)	17598
	Intervention terms-3 (Relaxation therapy related)	
S7	"relaxation therapy" or "relaxation technique" or meditation or "music therapy" or "verbal protocol" or "guided imagery"	17959
	Intervention terms-4 (Mental Health interventions related)	
S8	"psychosocial N30 maternal" or ("maternal mental health" N30 postnatal) or "maternal depression" or "postpartum depression" or ("peer support" N30 maternal) or "peer-support" or "social support" or "Postpartum anxiety" or "Postpartum psychological distress" or "Postnatal Debriefing" or " postnatal Psychoeducation" or "postnatal Psychotherapy"	59700
	Intervention terms-5 (KMC related)	
S9	"kangaroo mother care" or "KMC" or "kangaroo care" or "skin to skin care" or "Skin-to-Skin Contact"	2136
	Intervention terms-6 (Women empowerment related)	
S10	"empowerment" or "decision making" or "autonomy" or "women time use"	193719
	Intervention terms-7 (Cash Transfer related)	
S11	"social safety net" or "cash transfers" or "child support grant" or "social transfer" or "social assistance" or "Maternity Benefit Programme" or "Direct Benefit Transfers"	2345
S12	AND S5	1459
S13	S4 AND S6	678
S14	S4 AND S7	58
S15	S4 AND S8	428
S16	S4 AND S9	427
S17	S4 AND S10	349
S18	S4 AND S11	21
S19	S12 OR S13 OR S14 OR S15 OR S16 OR S17 OR S18	3079
S20	S12 OR S13 OR S14 OR S15 OR S16 OR S17 OR S18	2910
	Population and Intervention terms	
S21	S1 AND S20	982

Database 3: Cochrane Library

S	Terms	Hits #
	Filters used: Year: 2000 to 2021	

S	Terms	Hits #
	Population terms	
#1	(maternal or mother* or lactat* or parent* or famil* or postnatal or carer or caregiver or father or grandmother) AND (wasting or wasted or WLZ or WHZ or underweight or WAZ or "low mid upper arm circumference" or MUAC or preterm or "small size at birth" or "small for gestational age at birth" or "less than 37 weeks gestation" or "low birth weight" or "losing weight" or "infant near/5 six months" or "infant near/5 6 month"):ti,ab,kw (Word variations have been searched)	9233
#2	#1 NOT (antenatal or "ante-natal"):ti,ab,kw	8137
	Intervention terms-1 (Maternal Nutrition supplementation related)	
#3	supplement* or "folate" or "folic acid" or "iron" or "iron folic acid" or "IFA" or "calcium" or "iodine" or "vitamin D" or "zinc" or "multiple micronutrients" or "multiple micronutrients" or "multiple micro-nutrients" or "multi nutrient" or "multi-nutrient" or "multi-vitamin" or "multi-mineral" or "*nutrient supplement" or "LNS" or "balanced energy protein" or "macronutrient" or "*unsaturated fatty acid" or *PUFA or *LCPUFA or Biofortifi* or iodides or "Ergocalciferols" or "docosahexaenoic acid" or DHA or choline or "omega-3 fatty acid" (Word variations have been searched)	148077
	Intervention terms-2 (Education intervention related)	
#4	("nutrition education near/30 breastfeeding" or "nutrition counselling near/20 breastfeeding" or "breast feeding" or "breast-feeding" or "complementary feeding" or "Postnatal NEAR/20 education" or ((training* or promotion*) near/20 (breastfeeding or "breast-feeding" or feeding or postnatal or "post-natal"))) (Word variations have been searched)	7105
	Intervention terms-3 (Relaxation therapy related)	
#5	("relaxation therapy" or "relaxation technique" or "meditation" or "music therapy" or "verbal protocol" or "guided imagery" or "relaxation lighting" or "mind stress-releasing therapy" or "imagery psychotherapy") (Word variations have been searched)	9142
	Intervention terms-4 (Mental Health interventions related)	
#6	"psychosocial near/30 maternal" or ("maternal mental health" near/30 postnatal) or "maternal depression" or "postpartum depression" or ("peer support" near/30 maternal) or "peer-support" or "social support" or "Postpartum anxiety" or "Postpartum psychological distress" or "Postnatal Debriefing" or " postnatal Psychoeducation" or "postnatal Psychotherapy" (Word variations have been searched)	10387
	Intervention terms-5 (KMC related)	
#7	"kangaroo mother care" or "KMC" or "kangaroo care" or "skin to skin care" or "Skin-to-Skin Contact" (Word variations have been searched)	946
	Intervention terms-6 (Women empowerment related)	
#8	"empowerment" or "decision making" or "autonomy" or "women time use" (Word variations have been searched)	20853
	Intervention terms-7 (Cash Transfer related)	
#9	"social safety net" or "cash transfers" or "child support grant" or "social transfer" or "social assistance" or "Maternity Benefit Programme" or "Direct Benefit Transfers" or "public policy" (Word variations have been searched)	891
#10	#2 AND #3	2117

S	Terms	Hits #
#11	#2 AND #4	576
#12	#2 AND #5	73
#13	#2 AND #6	174
#14	#2 AND #7	419
#15	#2 AND #8	176
#16	#2 AND #9	29
	Population and Intervention terms	
#17	#10 OR #11 OR #12 OR #13 OR #14 OR #15 OR #16 with Publication Year from 2000 to 2021, in Trials (Word variations have been searched)	2839

Database 4: Embase

S	Terms	Hits #
	Filters used: Cochrane Highly Sensitive Search Strategy for identifying controlled trials in Embase: (2018 revision); Ovid format https://training.cochrane.org/handbook/current/chapter-04-technical-supplement-searching-and-selecting-studies#_Ref19195422<br>Year: 2000 to 2021	
	Population terms	
1	(maternal or mother* or father* or postnatal or lactat* or carer or caregiver or grandmother or carer or postpartum).ti,ab,hw.	1113402
2	(wasting or wasted or WLZ or WHZ or underweight or WAZ or "low mid upper arm circumference" or MUAC or preterm or "small size at birth" or "small for gestational age at birth" or "less than 37 weeks gestation" or "low birth weight" or "losing weight" or "infant near/5 six months" or "infant near/5 6 month").ti,ab.	183192
3	(1 and 2) not (antenatal or "ante natal").ti,ab.	55494
	Intervention terms-1 (Maternal Nutrition supplementation related)	
4	("multiple micronutrients" or zinc or calcium or "folic acid" or folate or iodine or IFA or supplement* or micronutrient or vitamin or mineral or "balanced energy protein" or "Polyunsaturated fatty acid" or PUFA or LCPUFA or iodides or "docosahexaenoic acid" or "omega-3 fatty acid").mp.	1990491
	Intervention terms-2 (Education intervention related)	
5	((education or counselling or training or promotion) adj50 (breastfeeding or complementary feeding or postnatal* or postpartum or feeding)).mp. [mp=title, abstract, heading word, drug trade name, original title, device manufacturer, drug manufacturer, device trade name, keyword, floating subheading word, candidate term word]	30927
	Intervention terms-3 (Relaxation therapy related)	
6	("relaxation therapy" or "relaxation technique" or "meditation" or "music therapy" or "verbal protocol" or "guided imagery").mp. [mp=title, abstract, heading word, drug trade name, original title, device manufacturer, drug manufacturer, device trade name, keyword, floating subheading word, candidate term word]	21088
	Intervention terms-4 (Mental Health interventions related)	
7	(((psychosocial or "psycho-social") adj20 maternal) or ("maternal mental health" adj30 postnatal) or "maternal depression" or "postpartum depression" or (("peer-support" or "social support") adj30 (mother* or maternal or father* or grandmother)) or "Postpartum anxiety" or "Postpartum psychological distress" or ((Psychoeducation or Psychotherap*) adj20 (postnatal or postpartum))).mp. [mp=title, abstract, heading word, drug trade name, original title, device manufacturer, drug manufacturer, device trade name, keyword, floating subheading word, candidate term word]	15550
	Intervention terms-5 (KMC related)	
8	("kangaroo mother care" or KMC or "kangaroo care" or "skin to skin care" or "Skin-to-Skin Contact").mp. [mp=title, abstract, heading word, drug trade name, original title, device manufacturer, drug manufacturer, device trade name, keyword, floating subheading word, candidate term word]	3211
	Intervention terms-6 (Women empowerment related)	

S	Terms	Hits #
9	(empowerment or autonomy or "household decision making" or "women time use").mp. [mp=title, abstract, heading word, drug trade name, original title, device manufacturer, drug manufacturer, device trade name, keyword, floating subheading word, candidate term word]	72272
	Intervention terms-7 (Cash Transfer related)	
10	("Social safety net" or "cash transfers" or "child support grant" or "Social transfer" or "social assistance" or "Maternity Benefit Programme" or "Direct Benefit Transfers").mp. [mp=title, abstract, heading word, drug trade name, original title, device manufacturer, drug manufacturer, device trade name, keyword, floating subheading word, candidate term word]	1768
11	3 and 4	5951
12	3 and 5	1199
13	3 and 6	62
14	3 and 7	746
15	3 and 8	708
16	3 and 9	154
17	3 and 10	24
18	11 or 12 or 13 or 14 or 15 or 16 or 17	8358
	Population and Intervention terms	
19	(Randomized controlled trial/ or Controlled clinical study/ or random*.ti,ab. or randomization/ or intermethod comparison/ or placebo.ti,ab. or (compare or compared or comparison).ti. or ((evaluated or evaluate or evaluating or assessed or assess) and (compare or compared or comparing or comparison)).ab. or (open adj label).ti,ab. or ((double or single or doubly or singly) adj (blind or blinded or blindly)).ti,ab. or double blind procedure/ or parallel group*1.ti,ab. or (crossover or cross over).ti,ab. or ((assign* or match or matched or allocation) adj5 (alternate or group*1 or intervention*1 or patient*1 or subject*1 or participant*1)).ti,ab. or (assigned or allocated).ti,ab. or (controlled adj7 (study or design or trial)).ti,ab. or (volunteer or volunteers).ti,ab. or human experiment/ or trial.ti.) not (((random* adj sampl* adj7 ("cross section*" or questionnaire*1 or survey* or database*1)).ti,ab. not (comparative study/ or controlled study/ or randomi?ed controlled.ti,ab. or randomly assigned.ti,ab.)) or (Cross-sectional study/ not (randomized controlled trial/ or controlled clinical study/ or controlled study/ or randomi?ed controlled.ti,ab. or control group*1.ti,ab.)) or (((case adj control*) and random*) not randomi?ed controlled).ti,ab. or (Systematic review not (trial or study)).ti. or (nonrandom* not random*).ti,ab. or "Random field*".ti,ab. or (random cluster adj3 sampl*).ti,ab. or ((review.ab. and review.pt.) not trial.ti.) or ("we searched".ab. and (review.ti. or review.pt.)) or "update review".ab. or (databases adj4 searched).ab. or ((rat or rats or mouse or mice or swine or porcine or murine or sheep or lambs or pigs or piglets or rabbit or rabbits or cat or cats or dog or dogs or cattle or bovine or monkey or monkeys or trout or marmoset*1).ti. and animal experiment/) or (Animal experiment/ not (human experiment/ or human/)))	4784487
20	18 and 19	2407
21	limit 20 to yr="2000 - 2021"	2165

Database 5: Global Health

S	Terms	Hits #
	Filters used: Cochrane Highly Sensitive Search Strategy for identifying controlled trials in Embase: (2018 revision); Ovid format https://training.cochrane.org/handbook/current/chapter-04-technical-supplement-searching-and-selecting-studies#_Ref19195422<br>Year: 2000 to 2021	
	Population terms	
1	(maternal or mother* or father or postnatal or lactat* or caregiver or grandmother).ti,ab.	185659
2	(wasting or wasted or WLZ or WHZ or underweight or WAZ or "low mid upper arm circumference" or MUAC or preterm or "small size at birth" or "small for gestational age at birth" or "less than 37 weeks gestation" or "low birth weight" or "losing weight" or "infant near/5 six months" or "infant near/5 6 month").ti,ab,hw.	43687
3	(1 and 2) not (antenatal or "ante natal").mp. [mp=abstract, title, original title, broad terms, heading words, identifiers, cabicodes]	13936
	Intervention terms-1 (Maternal Nutrition supplementation related)	
4	(supplement* or folic acid or folate or iron or "iron folic acid" or "IFA" or calcium or iodine or "vitamin D" or vitaminD or zinc or micronutrient or vitamin or mineral or "balanced energy protein" or "Polyunsaturated fatty acid" or PUFA or LCPUFA or "omega-3 fatty acid").mp. [mp=abstract, title, original title, broad terms, heading words, identifiers, cabicodes]	488594
	Intervention terms-2 (Education intervention related)	
5	((education or counselling or training or promotion) adj30 (breastfeeding or "breast feeding" or complementary feeding or postnatal* or postpartum)).mp. [mp=abstract, title, original title, broad terms, heading words, identifiers, cabicodes]	6315
	Intervention terms-3 (Relaxation therapy related)	
6	(relaxation therapy or relaxation technique or meditation or music therapy or verbal protocol or guided imagery).mp. [mp=abstract, title, original title, broad terms, heading words, identifiers, cabicodes]	1292
	Intervention terms-4 (Mental Health interventions related)	
7	(((psychosocial or "psycho-social") adj20 maternal) or ("maternal mental health" adj20 postnatal) or "maternal depression" or "postpartum depression" or peer support or peer-support or social support or Postpartum anxiety or Postpartum psychological distress or postnatal Psychoeducation or postnatal Psychotherap*).mp. [mp=abstract, title, original title, broad terms, heading words, identifiers, cabicodes]	11732
	Intervention terms-5 (KMC related)	
8	("kangaroo mother care" or KMC or "kangaroo care" or "skin to skin care" or "Skin-to-Skin Contact").mp. [mp=abstract, title, original title, broad terms, heading words, identifiers, cabicodes]	690
	Intervention terms-6 (Women empowerment related)	
9	(empowerment or autonomy or "household decision making" or "women time use").mp. [mp=abstract, title, original title, broad terms, heading words, identifiers, cabicodes]	9717
	Intervention terms-7 (Cash Transfer related)	
10	("Social safety net" or "cash transfers" or "child support grant" or "Social transfer" or "social assistance" or "Maternity Benefit Programme" or "Direct	899

S	Terms	Hits #
	Benefit Transfers").mp. [mp=abstract, title, original title, broad terms, heading words, identifiers, cabicodes]	
11	3 and 4	2361
12	3 and 5	364
13	3 and 6	5
14	3 and 7	163
15	3 and 8	194
16	3 and 9	81
17	3 and 10	10
18	11 or 12 or 13 or 14 or 15 or 16 or 17	3018
19	limit 18 to yr="2000 - 2021"	2492
20	(Randomized controlled trial/ or Controlled clinical study/ or random*.ti,ab. or randomization/ or intermethod comparison/ or placebo.ti,ab. or (compare or compared or comparison).ti. or ((evaluated or evaluate or evaluating or assessed or assess) and (compare or compared or comparing or comparison)).ab. or (open adj label).ti,ab. or ((double or single or doubly or singly) adj (blind or blinded or blindly)).ti,ab. or double blind procedure/ or parallel group*1.ti,ab. or (crossover or cross over).ti,ab. or ((assign* or match or matched or allocation) adj5 (alternate or group*1 or intervention*1 or patient*1 or subject*1 or participant*1)).ti,ab. or (assigned or allocated).ti,ab. or (controlled adj7 (study or design or trial)).ti,ab. or (volunteer or volunteers).ti,ab. or human experiment/ or trial.ti.) not (((random* adj sampl* adj7 ("cross section*" or questionnaire*1 or survey* or database*1)).ti,ab. not (comparative study/ or controlled study/ or randomi?ed controlled.ti,ab. or randomly assigned.ti,ab.)) or (Cross-sectional study/ not (randomized controlled trial/ or controlled clinical study/ or controlled study/ or randomi?ed controlled.ti,ab. or control group*1.ti,ab.)) or (((case adj control*) and random*) not randomi?ed controlled).ti,ab. or (Systematic review not (trial or study)).ti. or (nonrandom* not random*).ti,ab. or "Random field*".ti,ab. or (random cluster adj3 sampl*).ti,ab. or ((review.ab. and review.pt.) not trial.ti.) or ("we searched".ab. and (review.ti. or review.pt.)) or "update review".ab. or (databases adj4 searched).ab. or ((rat or rats or mouse or mice or swine or porcine or murine or sheep or lambs or pigs or piglets or rabbit or rabbits or cat or cats or dog or dogs or cattle or bovine or monkey or monkeys or trout or marmoset*1).ti. and animal experiment/) or (Animal experiment/ not (human experiment/ or human/)))	579734
	Population and Intervention terms	
21	19 and 20	798

Database 6: Medline (PubMed)

S	Terms	Hits #
	Filters used: Limits RCT Filters applied: sensitivity- and precision-maximizing version (2008 revision); PubMed format<br>MEDLINE, from 2000/1/1 - 2021/1/1.	
	Population terms	
1	(maternal OR mother* OR lactat* OR parent* OR famil* OR postnatal OR carer OR caregiver OR father OR grandmother OR grandparents) AND	4623

S	Terms	Hits #
	(wasting OR wasted OR WLZ OR WHZ OR underweight OR WAZ OR low mid upper arm circumference OR MUAC OR preterm OR <37 weeks gestation OR low birth weight OR losing weight OR infant under six months)	
	Intervention terms-1 (Maternal Nutrition supplementation related)	
2	(supplement* OR folate OR folic acid OR iron OR iron folic acid OR IFA OR calcium OR iodine OR vitamin D OR zinc OR multiple micronutrients OR multiple micro-nutrients OR multi nutrient OR multi-nutrient OR multi-vitamin OR multi-mineral OR lipid-based nutrient supplement OR LNS OR balanced energy protein OR macronutrient OR poly-unsaturated fatty-acids OR PUFA OR long chain poly-unsaturated fatty-acids OR LCPUFA OR n-3 LCPUFA OR n-3 PUFA OR Biofortification OR docosahexaenoic acid OR choline OR omega-3 fatty acids) NOT Antenatal	49884
	Intervention terms-1 (Combined with OR Boolean operator)	
3	#9 (Maternal Nutrient Supplementation)<br>(((maternal OR mother* OR lactat* OR parent* OR famil* OR postnatal OR caregiver OR father OR grandmother OR grandparents) AND (wasting OR wasted OR WLZ OR WHZ OR underweight OR WAZ OR low mid upper arm circumference OR MUAC OR preterm OR <37 weeks gestation OR small size at birth OR small for gestational age at birth OR low birth weight OR losing weight OR infant under six months)) AND ((supplement* OR folate OR folic acid OR iron OR iron folic acid OR IFA OR calcium OR iodine OR vitamin D OR zinc OR multiple micronutrients OR multiple micro-nutrients OR multi nutrient OR multi-nutrient OR multi-vitamin OR multi-mineral OR lipid-based nutrient supplement OR LNS OR balanced energy protein OR macronutrient OR poly-unsaturated fatty-acids OR PUFA OR long chain poly-unsaturated fatty-acids OR LCPUFA OR n-3 LCPUFA OR n-3 PUFA OR Biofortification OR docosahexaenoic acid OR choline OR omega-3 fatty acids))) NOT (Antenatal) Filters: MEDLINE, from 2000 - 2021	7475
	Intervention terms-2 (Education intervention related)	
4	(nutrition counselling OR nutrition education OR breastfeeding OR breast feeding OR breast-feeding OR complementary feeding OR postnatal breastfeeding education) NOT Antenatal	8805
	Intervention terms-2 (Combined with OR Boolean operator)	
5	#13 (Education/counselling interventions)<br>(maternal OR mother* OR lactat* OR parent* OR famil* OR postnatal OR carer OR caregiver OR father OR grandmother OR grandparents) AND (wasting OR wasted OR WLZ OR WHZ OR underweight OR WAZ OR low mid upper arm circumference OR MUAC OR preterm OR <37 weeks gestation OR low birth weight OR losing weight OR infant under six months)) AND ((nutrition counselling OR nutrition education OR breastfeeding OR breast feeding OR breast-feeding OR complementary feeding OR postnatal breastfeeding education) NOT Antenatal Filters: MEDLINE, from 2000 - 2021	6889
	Intervention terms-3 (Relaxation therapy related)	
6	(relaxation therapy OR relaxation technique OR meditation OR music therapy OR verbal protocol OR guided imagery OR mind stress-releasing therapy OR relaxation lighting OR imagery psychotherapy) NOT Antenatal	5190
	Intervention terms-3 (Combined with OR Boolean operator)	

S	Terms	Hits #
7	#15 (relaxation therapy while breastfeeding)<br>((maternal OR mother* OR lactat* OR parent* OR famil* OR postnatal OR carer OR caregiver OR father OR grandmother OR grandparents) AND (wasting OR wasted OR WLZ OR WHZ OR underweight OR WAZ OR low mid upper arm circumference OR MUAC OR preterm OR <37 weeks gestation OR low birth weight OR losing weight OR infant under six months)) AND ((relaxation therapy OR relaxation technique OR meditation OR music therapy OR verbal protocol OR guided imagery OR mind stress-releasing therapy OR relaxation lighting OR imagery psychotherapy) NOT Antenatal Filters: MEDLINE, from 2000 - 2021	145
	Intervention terms-4 (Mental Health interventions related)	
8	(psychosocial OR maternal mental health OR maternal depression OR postpartum depression OR postnatal blues OR Post-traumatic stress disorder OR PTSD OR postpartum anxiety OR Peer support OR social support OR Postpartum psychological distress OR Puerperal depression OR Baby blues OR Postnatal Debriefing OR postnatal Psychoeducation OR postnatal Psychotherapy)	28959
	Intervention terms-4 (Combined with OR Boolean operator)	
9	#18 (psychosocial and psychological interventions)<br>((maternal OR mother* OR lactat* OR parent* OR famil* OR postnatal OR carer OR caregiver OR father OR grandmother OR grandparents) AND (wasting OR wasted OR WLZ OR WHZ OR underweight OR WAZ OR low mid upper arm circumference OR MUAC OR preterm OR <37 weeks gestation OR low birth weight OR losing weight OR infant under six months)) AND (psychosocial OR maternal mental health OR maternal depression OR postpartum depression OR postnatal blues OR Post-traumatic stress disorder OR PTSD OR postpartum anxiety OR Peer support OR social support OR Postpartum psychological distress OR Puerperal depression OR Baby blues OR Postnatal Debriefing OR postnatal Psychoeducation OR postnatal Psychotherapy) NOT Antenatal) Filters: MEDLINE, from 2000 - 2021	5732
	Intervention terms-5 (KMC related)	
10	(kangaroo mother care OR KMC OR kangaroo care OR skin to skin care OR skin-to-skin contact OR SSC) NOT Antenatal	4722
	Intervention terms-5 (Combined with OR Boolean operator)	
11	#20 (KMC interventions)<br>((maternal OR mother* OR lactat* OR parent* OR famil* OR postnatal OR carer OR caregiver OR father OR grandmother OR grandparents) AND (wasting OR wasted OR WLZ OR WHZ OR underweight OR WAZ OR low mid upper arm circumference OR MUAC OR preterm OR <37 weeks gestation OR low birth weight OR losing weight OR infant under six months)) AND (kangaroo mother care OR KMC OR kangaroo care OR skin to skin care OR skin-to-skin contact OR SSC) NOT Antenatal Filters: MEDLINE, from 2000 - 2021	888
	Intervention terms-6 (Women empowerment related)	
12	(decision making OR autonomy OR empowerment OR household decision making OR women time use) NOT Antenatal	29221
	Intervention terms-6 (Combined with OR Boolean operator)	

S	Terms	Hits #
13	#22 (Women's empowerment interventions)<br>((maternal OR mother* OR lactat* OR parent* OR famil* OR postnatal OR carer OR caregiver OR father OR grandmother OR grandparents) AND (wasting OR wasted OR WLZ OR WHZ OR underweight OR WAZ OR low mid upper arm circumference OR MUAC OR preterm OR <37 weeks gestation OR low birth weight OR losing weight OR infant under six months)) AND (decision making OR autonomy OR empowerment OR household decision making OR women time use) NOT Antenatal Filters: MEDLINE, from 2000 - 2021	5353
	Intervention terms-7 (Cash Transfer related)	
14	(social safety net OR social protection OR cash transfers OR child support grant OR social transfer OR social assistance) NOT Antenatal	6676
	Intervention terms-7 (Combined with OR Boolean operator)	
15	#24 (Interventions to address poverty and vulnerability)<br>((maternal OR mother* OR lactat* OR parent* OR famil* OR postnatal OR carer OR caregiver OR father OR grandmother OR grandparents) AND (wasting OR wasted OR WLZ OR WHZ OR underweight OR WAZ OR low mid upper arm circumference OR MUAC OR preterm OR <37 weeks gestation OR low birth weight OR losing weight OR infant under six months)) AND (social safety net OR social protection OR cash transfers OR child support grant OR social transfer OR social assistance) NOT Antenatal Filters: MEDLINE, from 2000 - 2021	2001
	Population and Intervention terms	
16	#9 OR #13 OR #15 OR #18 OR #20 OR #22 OR #24 AND (randomized controlled trial[pt] OR controlled clinical trial[pt] OR randomized[tiab] OR placebo[tiab] OR clinical trials as topic[mesh:noexp] OR randomly[tiab] OR trial[ti] NOT (animals[mh] NOT humans [mh])) from 2000/1/1 - 2021/1/1	3225

Database 7: Science Direct

S	Terms	#Hits
	Filters used: Subject areas included- Medicine & Dentistry, Social Sciences, Nursing and Health Professions, Psychology, Agricultural & Biological Sciences, Article type- Research articles, Conference Abstracts, Year: 2000-2021	
	Population terms	
1	(maternal OR lactating OR postnatal OR caregiver OR father) AND (wasting OR underweight OR preterm OR infant under six months)	48326
	Intervention terms-1 (Maternal Nutrition supplementation related)	
2	supplement OR folate OR iron OR zinc OR multiple micronutrients OR lipid-based nutrient supplement OR balanced energy protein OR poly-unsaturated fatty-acids	526958
	Intervention terms-1 (Combined with OR Boolean operator)	
3	#1 AND #2 Maternal Nutrition Supplementation<br>(maternal) AND (wasting OR infants under six months) AND (supplements OR folate OR multiple micronutrients OR balanced energy protein OR poly-unsaturated fatty-acids) NOT Antenatal Journal or book title: JOURNAL Year: 2000-2021	803

S	Terms	#Hits
	Intervention terms-2 (Education intervention related)	
4	(nutrition counselling OR nutrition education OR breastfeeding OR breast feeding OR breast-feeding OR complementary feeding OR postnatal breastfeeding education)	122392
	Intervention terms-2 (Combined with OR Boolean operator)	
5	#1 AND #3 (Education/counselling interventions)<br>(maternal) AND (wasting OR infants under six months) AND (nutrition counselling OR nutrition education OR breastfeeding OR complementary feeding OR postnatal breastfeeding education) NOT Antenatal Journal or book title: JOURNAL Year: 2000-2021	872
	Intervention terms-3 (Relaxation therapy related)	
6	(relaxation therapy OR relaxation technique OR meditation OR music therapy OR verbal protocol OR guided imagery OR mind stress-releasing therapy OR relaxation lighting OR imagery psychotherapy)	92208
	Intervention terms-3 (Combined with OR Boolean operator)	
7	#1 AND #4 (relaxation therapy while breastfeeding)<br>(maternal) AND (wasting OR infants under six months) AND (relaxation therapy while breastfeeding OR relaxation technique OR meditation OR mind stress-releasing therapy OR music therapy) NOT Antenatal Journal or book title: JOURNAL Year: 2000-2021	130
	Intervention terms-4 (Mental Health interventions related)	
8	(psychosocial OR maternal depression OR postpartum depression OR Post-traumatic stress disorder OR postpartum anxiety OR Peer support OR social support)	501256
	Intervention terms-4 (Combined with OR Boolean operator)	
9	#1 AND #5 (psychosocial and psychological interventions)<br>(maternal) AND (wasting OR infants under six months) AND (psychosocial OR postpartum depression OR Peer support) NOT Antenatal Journal or book title: JOURNAL Year: 2000-2021	663
	Intervention terms-5 (KMC related)	
10	(kangaroo mother care OR KMC OR kangaroo care OR skin to skin care OR skin-to-skin contact OR SSC)	192684
	Intervention terms-5 (Combined with OR Boolean operator)	
11	#1 AND #6 (KMC interventions)<br>(maternal) AND (wasting OR infants under six months) AND (kangaroo mother care OR KMC OR kangaroo care OR skin to skin care) NOT Antenatal Journal or book title: JOURNAL Year: 2000-2021	441
	Intervention terms-6 (Women empowerment related)	
12	(decision making OR autonomy OR empowerment OR household decision making OR women time use)	773453
	Intervention terms-6 (Combined with OR Boolean operator)	
13	#1 AND #7 (Women's empowerment interventions)<br>(maternal) AND (wasting OR infants under six months) AND (decision making w/15 autonomy OR empowerment) NOT Antenatal Journal or book title: JOURNAL Year: 2000-2021	211
	Intervention terms-7 (Cash Transfer related)	

S	Terms	#Hits
14	(social safety net OR social protection OR cash transfers OR child support grant OR social transfer OR social assistance OR Maternity Benefit Programme OR Direct Benefit Transfers OR public policy)	427674
	Intervention terms-7 (Combined with OR Boolean operator)	
15	#1 AND #8 (Interventions to address poverty and vulnerability)<br>(maternal) AND (wasting OR infants under six months) AND (social safety net OR social protection OR cash transfers OR social assistance) NOT Antenatal Journal or book title: JOURNAL Year: 2000-2021	585
16	Total results of the individual interventions altogether with Publication Year from 2000 to 2021, in ScienceDirect	3,705

Database 8: Scopus

S	Terms	Hits #
	Filters used: Subject area- Medicine, Nursing, Psychology, Social Sciences, Health Professions, Agricultural & Biological Sciences, Multidisciplinary Document type- Articles only included, Applied RCT filters (This filter is built by NUS Medical Library using MESH and EMTREE terms as Index terms and Keywords), Year: 2000- 2021	
	Population terms	
1	maternal OR mother* OR lactat* OR postnatal OR caregiver OR father OR grandparents AND wasting OR wasted OR wlz OR whz OR underweight OR waz OR (low mid upper arm circumference) OR muac OR preterm OR (<37 weeks gestation) OR (small size at birth) OR (small for gestational age at birth) OR (low birth weight) OR (losing weight) OR (infant under six months)	212913
	Intervention terms-1 (Maternal Nutrition supplementation related)	
2	supplement* OR folate OR iron OR (iron folic acid) OR calcium OR iodine OR (vitamin D) OR zinc OR (multiple micronutrients) OR (multi nutrient) OR (multi-vitamin) OR (multi-mineral) OR "lipid-based nutrient supplement" OR (balanced energy protein) OR (macronutrient) OR "poly-unsaturated fatty-acids" OR (long chain poly-unsaturated fatty-acids) OR n-3 AND lcpufa OR n-3 AND pufa OR (Dietary supplements) OR (omega-3 fatty acids)	45658
	Intervention terms-1 (Combined with OR Boolean operator)	
#1 AND #2	(maternal OR mother* OR postnatal OR caregiver AND wasting OR underweight OR preterm OR (infant under six months)) AND (supplement* OR folate OR (iron folic acid) OR calcium OR iodine OR (vitamin D) OR zinc OR (multiple micronutrients) OR (multi-vitamin) OR (multi-mineral) OR "lipid-based nutrient supplement" OR (balanced energy protein) OR (macronutrient) OR "poly-unsaturated fatty-acids" OR (long chain poly-unsaturated fatty-acids) OR n-3 AND lcpufa OR n-3 AND pufa OR (omega-3 fatty acids)) AND NOT antenatal	1020
	Intervention terms-2 (Education intervention related)	
4	(Nutrition counselling) OR (nutrition education) OR breastfeeding OR (breast feeding) OR breast-feeding OR (complementary feeding) OR (postnatal breastfeeding education)	160403
	Intervention terms-2 (Combined with OR Boolean operator)	

S	Terms	Hits #
#1 AND #4	(maternal OR mother* OR lactat* OR postnatal OR caregiver OR father W/ 15 wasting OR wasted OR underweight OR (low mid upper arm circumference) OR muac OR preterm OR (small size at birth) OR (small for gestational age at birth) OR (low birth weight) OR (losing weight) OR (infant under six months)) AND ((Nutrition counselling) OR (nutrition education) OR breastfeeding OR (breast feeding) OR breast-feeding OR (complementary feeding) OR (postnatal breastfeeding education))	73
	Intervention terms-3 (Relaxation therapy related)	
6	(relaxation therapy) OR (relaxation technique) OR meditation OR (music therapy) OR (verbal protocol) OR (guided imagery) OR (mind stress-releasing therapy) OR (relaxation lighting) OR (imagery psychotherapy)	113068
	Intervention terms-3 (Combined with OR Boolean operator)	
#1 AND #6	(maternal OR mother* OR lactat* OR postnatal OR caregiver OR father AND wasting OR wasted OR underweight OR (low mid upper arm circumference) OR muac OR preterm OR (infant under six months)) AND ((relaxation therapy) OR (relaxation technique) OR meditation OR (music therapy) OR (mind stress-releasing therapy) OR (relaxation lighting) OR (imagery psychotherapy)) AND NOT antenatal	578
	Intervention terms-4 (Mental Health interventions related)	
8	psychosocial OR (maternal mental health) OR (maternal depression) OR (postpartum depression) OR (postnatal blues) OR (Post-traumatic stress disorder) OR (PTSD) OR (postpartum anxiety) OR (Peer support) OR (social support) OR (Postpartum psychological distress) OR (Puerperal depression) OR (Baby blues) OR (Postnatal Debriefing) OR (postnatal psychoeducation) OR (postnatal Psychotherapy)	951263
	Intervention terms-4 (Combined with OR Boolean operator)	
#1 AND #8	(maternal OR mother* OR lactat* OR postnatal OR caregiver AND wasting OR underweight OR preterm OR (infant under six months)) AND ((maternal depression) OR (postpartum depression) W/15 (Peer support) OR (social support)) AND NOT antenatal	205
	Intervention terms-5 (KMC related)	
10	(kangaroo mother care) OR kmc OR (kangaroo care) OR (skin to skin care) OR (skin-to-skin contact) OR ssc	77632
	Intervention terms-5 (Combined with OR Boolean operator)	
#1 AND #10	(maternal OR mother* OR lactat* OR postnatal OR caregiver W/ 15 wasting OR underweight OR preterm OR (infant under six months)) AND ((kangaroo mother care) OR kmc OR (kangaroo care) OR (skin to skin care) OR (skin-to-skin contact)) AND NOT antenatal	12
	Intervention terms-6 (Women empowerment related)	
12	(decision making) OR autonomy OR empowerment OR (household decision making) OR (women time use)	1758994
	Intervention terms-6 (Combined with OR Boolean operator)	

<table>
<tr><th>S</th><th>Terms</th><th>Hits #</th></tr>
<tr><td>#1 AN D #12</td><td>(maternal OR mother* OR lactat* OR postnatal OR caregiver OR father OR grandparents W/ 15 wasting OR underweight OR waz OR (low mid upper arm circumference) OR muac OR preterm OR (<37 weeks gestation) OR (small size at birth) OR (small for gestational age at birth) OR (low birth weight) OR (losing weight) OR (infant under six months)) AND ((decision making) OR autonomy OR empowerment OR (household decision making) OR (women time use))</td><td>26</td></tr>
<tr><td></td><td>Intervention terms-7 (Cash Transfer related)</td><td></td></tr>
<tr><td>14</td><td>(social safety net) OR (social protection) OR (cash transfers) OR (child support grant) OR (social transfer) OR (social assistance) OR (Maternity Benefit Programme) OR (Direct Benefit Transfers) OR (public policy)</td><td>526762</td></tr>
<tr><td></td><td>Intervention terms-7 (Combined with OR Boolean operator)</td><td></td></tr>
<tr><td>#1 AN D #14</td><td>(maternal OR mother* OR lactat* OR postnatal OR caregiver OR father OR grandparents W/ 15 wasting OR wasted OR wlz OR whz OR underweight OR waz OR (low mid upper arm circumference) OR muac OR preterm OR (small size at birth) OR (small for gestational age at birth) OR (low birth weight) OR (losing weight) OR (infant under six months)) AND ((social safety net) OR (social protection) OR (cash transfers) OR (child support grant) OR (social transfer) OR (social assistance) OR (Maternity Benefit Programme) OR (Direct Benefit Transfers) OR (public policy))</td><td>7</td></tr>
<tr><td></td><td>Population and Intervention terms</td><td></td></tr>
<tr><td></td><td>(#1 AND #2) OR (#1 AND #4) OR (#1 AND #6) OR (#1 AND #8) OR (#1 AND #10) OR (#1 AND #12) OR (#1 AND #14) with Publication Year from 2000 to 2021, in Scopus database</td><td>1901</td></tr>
<tr><td colspan="3">With RCT FILTERS APPLIED</td></tr>
<tr><td colspan="3">((maternal OR mother* OR postnatal OR caregiver AND wasting OR underweight OR preterm OR (infant under six months)) AND (supplement* OR folate OR (iron folic acid) OR calcium OR iodine OR (vitamin D) OR zinc OR (multiple micronutrients) OR (multi-vitamin) OR (multi-mineral) OR "lipid-based nutrient supplement" OR (balanced energy protein) OR (macronutrient) OR "poly-unsaturated fatty-acids" OR (long chain poly-unsaturated fatty-acids) OR n-3 AND lcpufa OR n-3 AND pufa OR (omega-3 fatty acids)) AND NOT antenatal) OR ((maternal OR mother* OR lactat* OR postnatal OR caregiver OR father W/ 15 wasting OR wasted OR underweight OR (low mid upper arm circumference) OR muac OR preterm OR (small size at birth) OR (small for gestational age at birth) OR (low birth weight) OR (losing weight) OR (infant under six months)) AND ((Nutrition counselling) OR (nutrition education) OR breastfeeding OR (breast feeding) OR breast-feeding OR (complementary feeding) OR (postnatal breastfeeding education)))
OR ((maternal OR mother* OR lactat* OR postnatal OR caregiver OR father AND wasting OR wasted OR underweight OR (low mid upper arm circumference) OR muac OR preterm OR (infant under six months)) AND ((relaxation therapy) OR (relaxation technique) OR meditation OR (music therapy) OR (mind stress-releasing therapy) OR (relaxation lighting) OR (imagery psychotherapy)) AND NOT antenatal) OR ((maternal OR mother* OR lactat* OR postnatal OR caregiver AND wasting OR underweight OR preterm OR (infant under six months)) AND ((maternal depression) OR (postpartum depression) W/15 (Peer support) OR (social support)) AND NOT antenatal) OR ((maternal OR mother* OR lactat* OR postnatal OR caregiver W/ 15 wasting OR</td></tr>
</table>

S	Terms	Hits #
underweight OR preterm OR (infant under six months)) AND ((kangaroo mother care) OR kmc OR (kangaroo care) OR (skin to skin care) OR (skin-to-skin contact)) AND NOT antenatal) OR ((maternal OR mother* OR lactat* OR postnatal OR caregiver OR father OR grandparents W/ 15 wasting OR underweight OR waz OR (low mid upper arm circumference) OR muac OR preterm OR (<37 weeks gestation) OR (small size at birth) OR (small for gestational age at birth) OR (low birth weight) OR (losing weight) OR (infant under six months)) AND ((decision making) OR autonomy OR empowerment OR (household decision making) OR (women time use))) OR ((maternal OR mother* OR lactat* OR postnatal OR caregiver OR father OR grandparents W/ 15 wasting OR wasted OR wlz OR whz OR underweight OR waz OR (low mid upper arm circumference) OR muac OR preterm OR (small size at birth) OR (small for gestational age at birth) OR (low birth weight) OR (losing weight) OR (infant under six months)) AND ((social safety net) OR (social protection) OR (cash transfers) OR (child support grant) OR (social transfer) OR (social assistance) OR (Maternity Benefit Programme) OR (Direct Benefit Transfers) OR (public policy))) (INDEXTERMS ("clinical trials" OR "clinical trials as a topic" OR "randomized controlled trial" OR "Randomized Controlled Trials as Topic" OR "controlled clinical trial" OR "Controlled Clinical Trials" OR "random allocation" OR "Double-Blind Method" OR "Single-Blind Method" OR "Cross-Over Studies" OR "Placebos" OR "multicenter study" OR "double blind procedure" OR "single blind procedure" OR "crossover procedure" OR "clinical trial" OR "controlled study" OR "randomization" OR "placebo")) OR (TITLE-ABS-KEY (("clinical trials" OR "clinical trials as a topic" OR "randomized controlled trial" OR "Randomized Controlled Trials as Topic" OR "controlled clinical trial" OR "Controlled Clinical Trials as Topic" OR "random allocation" OR "randomly allocated" OR "allocated randomly" OR "Double-Blind Method" OR "Single-Blind Method" OR "Cross-Over Studies" OR "Placebos" OR "cross-over trial" OR "single blind" OR "double blind" OR "factorial design" OR "factorial trial"))) OR (TITLE-ABS (clinical AND trial* OR trial* OR rct* OR random* OR blind*)) AND (LIMIT-TO (SUBJAREA , "MEDI") OR LIMIT-TO (SUBJAREA , "NURS") OR LIMIT-TO (SUBJAREA , "AGRI") OR LIMIT-TO (SUBJAREA , "PSYC") OR LIMIT-TO (SUBJAREA , "MULT") OR LIMIT-TO (SUBJAREA , "SOCI") OR LIMIT-TO (SUBJAREA , "HEAL")) AND (LIMIT-TO (DOCTYPE , "ar")) AND (LIMIT-TO (PUBYEAR , 2021) OR LIMIT-TO (PUBYEAR , 2020) OR LIMIT-TO (PUBYEAR , 2019) OR LIMIT-TO (PUBYEAR , 2018) OR LIMIT-TO (PUBYEAR , 2017) OR LIMIT-TO (PUBYEAR , 2016) OR LIMIT-TO (PUBYEAR , 2015) OR LIMIT-TO (PUBYEAR , 2014) OR LIMIT-TO (PUBYEAR , 2013) OR LIMIT-TO (PUBYEAR , 2012) OR LIMIT-TO (PUBYEAR , 2011) OR LIMIT-TO (PUBYEAR , 2010) OR LIMIT-TO (PUBYEAR , 2009) OR LIMIT-TO (PUBYEAR , 2008) OR LIMIT-TO (PUBYEAR , 2007) OR LIMIT-TO (PUBYEAR , 2006) OR LIMIT-TO (PUBYEAR , 2005) OR LIMIT-TO (PUBYEAR , 2004) OR LIMIT-TO (PUBYEAR , 2003) OR LIMIT-TO (PUBYEAR , 2002) OR LIMIT-TO (PUBYEAR , 2001) OR LIMIT-TO (PUBYEAR , 2000))		
Final – 924		

Database 9: Web of Science

SR. No	Terms	Hits #
	Filters used: RCT Filters used by Cochrane ENT, Year: 2000-2021	
	Population terms	

SR. No	Terms	Hits #
1	ALL=((maternal OR mother* OR lactat* OR parent* OR famil* OR postnatal OR carer OR caregiver OR father OR grandmother OR grandparents) AND (wasting OR wasted OR WLZ OR WHZ OR underweight OR WAZ OR low mid upper arm circumference OR MUAC OR preterm OR <37 weeks gestation OR small size at birth OR small for gestational age at birth OR low birth weight OR losing weight OR infant under six months)) and Articles (Document Types) and Obstetrics Gynecology or Pediatrics or Public Environmental Occupational Health or Nutrition Dietetics or Medicine General Internal or Endocrinology Metabolism or Multidisciplinary Sciences or Reproductive Biology or Nursing (Web of Science Categories) and Forum Of Nutrition or Annals Of The New York Academy Of Sciences Series or World Review Of Nutrition And Dietetics or Advances In Experimental Medicine And Biology or Annals Of The New York Academy Of Sciences (Exclude – Book Series Titles)	42560
	Intervention terms-1 (Maternal Nutrition supplementation related)	
2	ALL=((supplement* OR folate OR iron OR iron folic acid OR IFA OR calcium OR iodine OR vitamin D OR zinc OR multiple micronutrients OR multiple micro-nutrients OR multi nutrient OR multi-vitamin OR multi-mineral OR lipid-based nutrient supplement OR balanced energy protein OR macronutrient OR poly-unsaturated fatty-acids OR PUFA OR long chain poly-unsaturated fatty-acids OR LCPUFA OR n-3 LCPUFA OR n-3 PUFA OR omega-3 fatty acids)) and Articles (Document Types) and Food Science Technology or Nutrition Dietetics (Web of Science Categories) and Annals Of The New York Academy Of Sciences or Annals Of The New York Academy Of Sciences or World Review Of Nutrition And Dietetics or Advances In Experimental Medicine And Biology or Forum Of Nutrition or Acs Symposium Series or Contributions To Nephrology or Annals Of The New York Academy Of Sciences Series or Who Technical Report Series or Advances In Biochemical Engineering Biotechnology or Advances In Nutritional Research (Exclude – Book Series Titles)	82697
	Intervention terms-1 (Combined with OR Boolean operator)	
3	#1 AND #2 Maternal Nutrition Supplementation (TS=(maternal OR mother* OR lactat* OR parent* OR famil*OR postnatal OR caregiver OR father OR grandparents) AND TS=(wasting OR underweight OR infant under six months) AND TS=(supplement* near/15 folate OR iron folic acid OR calcium OR iodine OR vitamin D OR zinc OR multiple micronutrients OR multi-vitamin OR multi-mineral OR lipid-based nutrient supplement OR balanced energy protein OR macronutrient OR poly-unsaturated fatty-acids OR long chain poly-unsaturated fatty-acids OR omega-3 fatty acids)) NOT ALL=(Antenatal)	405
	Intervention terms-2 (Education intervention related)	
4	ALL=((nutrition counselling OR nutrition education OR breastfeeding OR breast feeding OR breast-feeding OR complementary feeding OR postnatal breastfeeding education)) and Articles (Document Types) and Public Environmental Occupational Health or Nutrition Dietetics or Pediatrics or Food Science Technology or Endocrinology Metabolism or Obstetrics Gynecology or Medicine General Internal or Nursing (Web of Science	60863

SR. No	Terms	Hits #
	Categories) and Articles (Document Types) and Advances In Experimental Medicine And Biology or World Review Of Nutrition And Dietetics or Annals Of The New York Academy Of Sciences or Forum Of Nutrition or Advances In Nutritional Research or Annals Of The New York Academy Of Sciences Series or Contributions To Nephrology (Exclude – Book Series Titles)	
	Intervention terms-2 (Combined with OR Boolean operator)	
5	#1 AND #3 (Education/counselling interventions) ((TS=(maternal OR mother* OR lactat* OR postnatal OR caregiver OR father OR grandparents)) AND (TS=(wasting OR infant under six months)) AND (TS=(nutrition counselling OR nutrition education OR breastfeeding OR complementary feeding OR postnatal breastfeeding education))) NOT ALL=(Antenatal)	318
	Intervention terms-3 (Relaxation therapy related)	
6	ALL=((relaxation therapy OR relaxation technique OR meditation OR music therapy OR verbal protocol OR guided imagery OR mind stress-releasing therapy OR relaxation lighting OR imagery psychotherapy)) and Articles (Document Types) and Psychiatry or Psychology Clinical or Multidisciplinary Sciences or Integrative Complementary Medicine or Psychology Multidisciplinary or Psychology or Nursing or Medicine General Internal or Psychology Experimental or Public Environmental Occupational Health or Medicine Research Experimental or Health Care Sciences Services or Pediatrics or Psychology Applied or Psychology Developmental or Social Sciences Interdisciplinary or Biology or Psychology Educational or Psychology Social or Social Sciences Biomedical or Nutrition Dietetics or Psychology Biological or Health Policy Services or Sociology (Web of Science Categories)	12580
	Intervention terms-3 (Combined with OR Boolean operator)	
7	#1 AND #3 (relaxation therapy while breastfeeding) (((TS=(maternal OR mother* OR lactat* OR postnatal OR caregiver OR father OR grandparents) AND (TS=(wasting OR underweight OR preterm OR infant under six months)))AND (TS= (relaxation therapy OR relaxation technique OR meditation OR music therapy OR verbal protocol OR guided imagery OR mind stress-releasing therapy OR relaxation lighting OR imagery psychotherapy)))) NOT ALL=(Antenatal)	74
	Intervention terms-4 (Mental Health interventions related)	
8	ALL=(psychosocial OR maternal mental health OR maternal depression OR postpartum depression OR postnatal blues OR Post-traumatic stress disorder OR PTSD OR postpartum anxiety OR Peer support OR social support OR Postpartum psychological distress OR Puerperal depression) and Articles (Document Types)	486463
	Intervention terms-4 (Combined with OR Boolean operator)	
9	#1 AND #4 (psychosocial and psychological interventions) (((TS=(maternal OR mother* OR lactat* OR postnatal OR caregiver OR father Or grandparents) AND ((TS=(wasting OR infant under six months)) AND (TS= (psychosocial OR maternal mental health OR maternal depression OR postpartum depression OR postnatal blues OR Post-traumatic stress disorder OR PTSD OR postpartum anxiety OR Peer	79

SR. No	Terms	Hits #
	support OR social support OR Postpartum psychological distress OR Puerperal depression))))) NOT ALL=(Antenatal))	
	Intervention terms-5 (KMC related)	
10	(kangaroo mother care OR KMC OR kangaroo care OR skin to skin care OR skin-to-skin contact OR SSC) Filters: Clinical Trial, Randomized Controlled Trial, MEDLINE, from 2000/1/1 - 2021/1/1	56463
	Intervention terms-5 (Combined with OR Boolean operator)	
11	#1 AND #5 (KMC interventions)<br>((TS=(maternal OR mother* OR lactat* OR postnatal OR caregiver OR father OR grandparents) AND (TS=(wasting OR underweight OR infant under six months)) AND (TS=((kangaroo mother care OR KMC OR kangaroo care OR skin to skin care OR skin-to-skin contact OR SSC))))) NOT ALL=(Antenatal)	11
	Intervention terms-6 (Women empowerment related)	
12	((TS=(decision making OR autonomy OR empowerment OR household decision making OR women time use)))	469038
	Intervention terms-6 (Combined with OR Boolean operator)	
13	#1 AND #6 (Women's empowerment interventions)<br>((((TS=(maternal OR mother* OR lactat* OR postnatal OR caregiver OR father OR grandparents) AND (TS=(wasting OR underweight OR infant under six months))) AND ((TS=(decision making OR autonomy OR empowerment OR household decision making OR women time use))))) NOT ALL=(Antenatal))	176
	Intervention terms-7 (Cash Transfer related)	
14	ALL=((social safety net OR social protection OR cash transfers OR child support grant OR social transfer OR social assistance OR Maternity Benefit Programme OR Direct Benefit Transfers OR public policy))	320459
	Intervention terms-7 (Combined with OR Boolean operator)	
15	#1 AND #9 (Interventions to address poverty and vulnerability)<br>((TS=(maternal OR mother* OR lactat* OR postnatal OR caregiver OR father OR grandparents) AND ((TS=((wasting OR underweight OR infant under six months)))) AND ((TS =((social safety net OR social protection OR cash transfers OR child support grant OR social transfer OR social assistance OR Maternity Benefit Programme OR Direct Benefit Transfers OR public policy))))) NOT ALL=(Antenatal))	75
	Population and Intervention terms	
16	(#1 OR #2 OR #3 OR #4 OR #5 OR #6 OR #7) AND TS=(randomised OR randomized OR randomisation OR randomisation OR placebo* OR (random* AND (allocat* OR assign*)) OR (blind* AND (single OR double OR treble OR triple))) from 2000-2021 in Web Of Science	166

Other sources: BIOSIS Previews

S	Terms	Hits #
	Filters used: Date range: 2000/01/01-2021/01/01	
	Population terms	
1	TS=(((maternal OR mother* OR lactat* OR parent* OR famil* OR postnatal OR carer OR caregiver OR father OR grandmother OR grandparents) AND (wasting OR wasted OR WLZ OR WHZ OR underweight OR WAZ OR low mid upper arm circumference OR MUAC OR preterm OR <37 weeks gestation OR small size at birth OR small for gestational age at birth OR low birth weight OR losing weight OR infant under six months)))	
	Intervention terms-1 (Maternal Nutrition supplementation related)	
2	TS=(((supplement* OR folate OR iron OR iron folic acid OR IFA OR calcium OR iodine OR vitamin D OR zinc OR multiple micronutrients OR multiple micro-nutrients OR multi nutrient OR multi-vitamin OR multi-mineral OR lipid-based nutrient supplement OR balanced energy protein OR macronutrient OR poly-unsaturated fatty-acids OR PUFA OR long chain poly-unsaturated fatty-acids OR LCPUFA OR n-3 LCPUFA OR n-3 PUFA OR omega-3 fatty acids)))	32,486
	Intervention terms-1 (Combined with OR Boolean operator)	
	(((TS=(maternal OR mother* OR lactat* OR parent* OR famil*OR postnatal OR caregiver OR father OR grandparents) AND TS=(wasting OR underweight OR infant under six months) AND TS=(supplement* near/15 folate OR iron folic acid OR calcium OR iodine OR vitamin D OR zinc OR multiple micronutrients OR multi-vitamin OR multi-mineral OR lipid-based nutrient supplement OR balanced energy protein OR macronutrient OR poly-unsaturated fatty-acids OR long chain poly-unsaturated fatty-acids OR omega-3 fatty acids)))) NOT TS=(Antenatal)	10,29,820
	Intervention terms-2 (Education intervention related)	
4	TS=(((nutrition counselling OR nutrition education OR breastfeeding OR breast feeding OR breast-feeding OR complementary feeding OR postnatal breastfeeding education)))	301
	Intervention terms-2 (Combined with OR Boolean operator)	
	((((TS=(maternal OR mother* OR lactat* OR postnatal OR caregiver OR father OR grandparents)) AND (TS=(wasting OR infant under six months)) AND (TS=(nutrition counselling OR nutrition education OR breastfeeding OR complementary feeding OR postnatal breastfeeding education))))) NOT TS=(Antenatal)	39,449
	Intervention terms-3 (Relaxation therapy related)	
6	TS=(((relaxation therapy OR relaxation technique OR meditation OR music therapy OR verbal protocol OR guided imagery OR mind stress-releasing therapy OR relaxation lighting OR imagery psychotherapy)))	220
	Intervention terms-3 (Combined with OR Boolean operator)	
	(((((TS=(maternal OR mother* OR lactat* OR postnatal OR caregiver OR father OR grandparents) AND (TS=(wasting OR underweight OR preterm OR infant under six months)))AND (TS= (relaxation therapy OR relaxation technique OR meditation OR music therapy OR verbal protocol OR guided imagery OR mind stress-releasing therapy OR relaxation lighting OR imagery psychotherapy)))))) NOT TS=(Antenatal)	43,151
	Intervention terms-4 (Mental Health interventions related)	

S	Terms	Hits #
8	TS=((psychosocial OR maternal mental health OR maternal depression OR postpartum depression OR postnatal blues OR Post-traumatic stress disorder OR PTSD OR postpartum anxiety OR Peer support OR social support OR Postpartum psychological distress OR Puerperal depression))	27
	Intervention terms-4 (Combined with OR Boolean operator)	
	(((((TS=(maternal OR mother* OR lactat* OR postnatal OR caregiver OR father Or grandparents) AND ((TS=(wasting OR infant under six months)) AND (TS= (psychosocial OR maternal mental health OR maternal depression OR postpartum depression OR postnatal blues OR Post-traumatic stress disorder OR PTSD OR postpartum anxiety OR Peer support OR social support OR Postpartum psychological distress OR Puerperal depression)))))))) NOT TS=(Antenatal)	98,053
	Intervention terms-5 (KMC related)	
10	TS=((kangaroo mother care OR KMC OR kangaroo care OR skin to skin care OR skin-to-skin contact OR SSC))	42
	Intervention terms-5 (Combined with OR Boolean operator)	
	(((((TS=(maternal OR mother* OR lactat* OR postnatal OR caregiver OR father OR grandparents) AND (TS=(wasting OR underweight OR infant under six months)) AND (TS=((kangaroo mother care OR KMC OR kangaroo care OR skin to skin care OR skin-to-skin contact OR SSC))))))) NOT TS=(Antenatal)	19,701
	Intervention terms-6 (Women empowerment related)	
12	(((TS=(decision making OR autonomy OR empowerment OR household decision making OR women time use))))	4
	Intervention terms-6 (Combined with OR Boolean operator)	
	((((((TS=(maternal OR mother* OR lactat* OR postnatal OR caregiver OR father OR grandparents) AND (TS=(wasting OR underweight OR infant under six months))) AND ((TS=(decision making OR autonomy OR empowerment OR household decision making OR women time use)))))))) NOT TS=(Antenatal)	1,35,864
	Intervention terms-7 (Cash Transfer related)	
14	TS=(((social safety net OR social protection OR cash transfers OR child support grant OR social transfer OR social assistance OR Maternity Benefit Programme OR Direct Benefit Transfers OR public policy)))	72
	Intervention terms-7 (Combined with OR Boolean operator)	
15	((((TS=(maternal OR mother* OR lactat* OR postnatal OR caregiver OR father OR grandparents) AND ((TS=((wasting OR underweight OR infant under six months)))) AND ((TS =((social safety net OR social protection OR cash transfers OR child support grant OR social transfer OR social assistance OR Maternity Benefit Programme OR Direct Benefit Transfers OR public policy)))))))) NOT TS=(Antenatal)	54,079
	Population and Intervention terms	
16	((#3 OR #5 OR #7 OR #9 OR #11 OR #13 OR #15))	67
	Total	645

Other sources: ISRCTN

Search #	Terms	Hits #
1	Postnatal maternal interventions	74
2	maternal intervention AND (supplement OR education OR KMC OR women empowerment OR social safety)	18

Other sources: WHO ICTRP

Search #	Terms	Hits #
1	(maternal OR mother OR caregiver OR postnatal) AND (supplement* OR KMC OR education OR relaxation OR psychological OR women empowerment OR social safety)	1122

Other sources: Clinicaltrial.gov

Search #	Terms	Hits #
1	(maternal OR mother OR caregiver OR postnatal)	3804
2	(maternal OR mother OR caregiver OR postnatal) AND (supplement* OR education OR relaxation therapy OR psychosocial OR KMC OR women empowerment OR social safety)	300
