# Supplementary material for: Effectiveness of Postnatal Maternal or Caregiver Interventions on Outcomes among Infants under Six Months with Growth Faltering: A Systematic Review
Source: Nutrients. 2024 Mar 14;16(6):837. doi: 10.3390/nu16060837 (PMC10974267; doi:10.3390/nu16060837)
Supplement: Supplementary file 1 [file nutrients-16-00837-s001.zip › S1 Table Rrisk of Bias_FinalProofread-author_13032024.pdf]

Risk of bias for RCTs

Sr. No.	Study	Outcome	Outcome type (as defined by guideline group members)	RoB (A-B-C-D-E-F)
	Intervention: breastfeeding counselling or education			
1	Ahmadi (2016) [24]	Weight at 1 m	important (but not critical)	+ + + + + +
2		Weight at 2 m	important (but not critical)	+ + + + + +
3		Weight at 3 m	low importance	+ + + + + +
4	Edraki (2015) [28]	Weight at 1 m	important (but not critical)	? + + + + ?
5		Weight at 2 m	important (but not critical)	? + + + + ?
6		Weight at 3 m	low importance	? + + + + ?
7		Weight at 6 m	low importance	? + + + + ?
8		Length at 1 m	important (but not critical)	? + + + + ?
9		Length at 2 m	low importance	? + + + + ?
10		Length at 3 m	low importance	? + + + + ?
11		Length at 6 m	low importance	? + + + + ?
12		HC at 1 m	low importance	? + + + + ?
13		HC at 2 m	low importance	? + + + + ?
14		HC at 3 m	low importance	? + + + + ?
15		HC at 6 m	low importance	? + + + + ?
16	Thakur (2012) [33]	Weight at 1 m	important (but not critical)	? ? + + ? ?
17		Weight at 2 m	important (but not critical)	? ? + + ? ?
18		Length at 1 m	important (but not critical)	? ? + + ? ?
19		Length at 2 m	low importance	? ? + + ? ?
20	Agarsada (2005) [27]	WAZ at 6 m	low importance	+ + + + ? ?
	Intervention: maternal nutrition supplementation			
21	Marc (2020) [35]	Weight by 36 wks PMA	important (but not critical)	+ + + + + +
22		Mortality by 36 wks PMA	important (but not critical)	+ + + + + +
	Intervention: mental health			
23	Seiiedi-Biarag (2021) [36]	Weight at 2 m	important (but not critical)	+ + + + + +
24		Length at 2 m	important (but not critical)	+ + + + + +
25		HC at 2 m	important (but not critical)	+ + + + + +
	Intervention: relaxation therapy			
26	Menke (2021) [37]	Weight at 3 m	important (but not critical)	? ? + + ? ?
27		Length at 3 m	important (but not critical)	? ? + + ? ?
28		HC at 3 m	important (but not critical)	? ? + + ? ?
	Intervention: cash transfer			
29	Andrews (2020) [38]	Child development score at 3 m	critical	- + + - + -
30		Change in weight z scores	important (but not critical)	- + - + + -
31		Change in HC z score	important (but not critical)	- + - + + -
32		Readmission by 3 m	important (but not critical)	- + - - + -

A: Bias arising from the randomisation process, B: Bias due to deviations from intended interventions; C: Bias due to missing outcome data; D: Bias in the measurement of the outcome; E: Bias in selcetion of the reported result; F: Overall risk of bias; HC: head circumference; m: month; PMA: postmenstural age; wks: weeks

Risk of bias for NRCTs

Sr. No.	Study	Outcome	ROBINS-I (overall)
	Intervention: breastfeeding counselling or education		
1	Eun hye (2020) [29]	Weight at 1 m	Serious
2		Length at 1 m	Serious
3		HC at 1 m	Serious
4		Readmission by 2 m	Serious
5	Gholami (2021) [30]	Readmission by 2 m	Moderate
6	Moudi (2017) [32]	Weight at 1 m	Moderate
7		Weight at 2 m	Moderate
	Intervention: maternal nutrition supplementation		
8	de Figueiredo (2010) [34]	Weight by 36 wks PMA	Critical
9		Length by 36 wks PMA	Critical
10		HC by 36 wks PMA	Critical

HC: head circumference; m: month; PMA: postmenstural age; wks: weeks
