# Supplementary material for: Effectiveness of Postnatal Maternal or Caregiver Interventions on Outcomes among Infants under Six Months with Growth Faltering: A Systematic Review
Source: Nutrients. 2024 Mar 14;16(6):837. doi: 10.3390/nu16060837 (PMC10974267; doi:10.3390/nu16060837)
Supplement: Supplementary file 1 [file nutrients-16-00837-s001.zip › S2 File Suppl Results_FinalProofread-author_13032024.pdf]

Supplementary results

Outcomes other than critical and important – low importance for guideline development

1) Effect of breastfeeding counselling or education versus standard care

Weight (g) at three and six months

Effect of breastfeeding counselling/education on weight (g) at 3 and 6 months (based on RCTs)

Study	Outcome	Participants	Mean Difference (95% CI)	RoB (A-B-C-D-E-F)
Ahmadi (2016) [24]	Weight at 3 m	124	598.02 [346.30, 849.74]	+ + + + + +
Edraki (2015) [28]	Weight at 3 m	59	346.80 [-83.40, 777.00]	? + + + + ?
	Weight at 6 m	57	840.60 [307.27, 1373.93]	? + + + + ?

A: Bias arising from the randomisation process, B: Bias due to deviations from intended interventions; C: Bias due to missing outcome data; D: Bias in the measurement of the outcome; E: Bias in selcetion of the reported result; F: Overall risk of bias

Length (cm) at two, three and six months

Effect of breastfeeding counselling/education on length (cm) at 2, 3, and 6 month (based on RCTs)

Study	Outcome	Participants	Mean Difference (95% CI)	RoB (A-B-C-D-E-F)
Edraki (2015) [28]	Length at 2 m	60	-2.39 [-5.64, 0.86]	? + + + + ?
Thakur (2012) [33]	Length at 2 m	184	1.50 [1.08, 1.92]	? ? + + ? ?
Edraki (2015) [28]	Length at 3 m	59	-2.32 [-5.69, 1.05]	? + + + + ?
	Length at 6 m	57	-2.25 [-5.83, 1.33]	? + + + + ?

A: Bias arising from the randomisation process, B: Bias due to deviations from intended interventions; C: Bias due to missing outcome data; D: Bias in the measurement of the outcome; E: Bias in selcetion of the reported result; F: Overall risk of bias

Head circumference (cm) at one month

Effect of breastfeeding counselling/education on HC (cm) at 1 month (based on RCT)

Study	Participants	Mean Difference (95% CI)	RoB (A-B-C-D-E-F)
Edraki (2015) [28]	60	-0.52 [-1.60, 0.56]	? + + + + ?

A: Bias arising from the randomisation process, B: Bias due to deviations from intended interventions; C: Bias due to missing outcome data; D: Bias in the measurement of the outcome; E: Bias in selcetion of the reported result; F: Overall risk of bias

Effect of breastfeeding counselling/education on HC (cm) at 1 month (based on NRCT)

Study	Participants	Mean Difference (95% CI)	ROBINS-I (overall)
Eun Hye (2020) [29]	53	0.70 [0.10, 1.30]	Serious

Head circumference (cm) at two, three, and six months

Effect of breastfeeding counselling/education on HC (cm) at 2, 3, and 6 months (based on RCT)

Study	Outcome	Participants	Mean Difference (95% CI)	RoB (A-B-C-D-E-F)
Edraki (2015) [28]	HC at 2 m	60	-0.76 [-1.96, 0.44]	? + + + + ?
	HC at 3 m	59	-0.71 [-2.02, 0.60]	? + + + + ?
	HC at 6 m	57	0.20 [-1.52, 1.92]	? + + + + ?

A: Bias arising from the randomisation process, B: Bias due to deviations from intended interventions; C: Bias due to missing outcome data; D: Bias in the measurement of the outcome; E: Bias in selcetion of the reported result; F: Overall risk of bias

WAZ at six months

Effect of breastfeeding counselling on WAZ at 6 months (based on RCT)

Study	Participants	Mean Difference (95% CI)	RoB (A-B-C-D-E-F)
Agarsada (2005) [27]	119	-2.02 [-2.33, -1.71]	+ + + + ? ?

A: Bias arising from the randomisation process, B: Bias due to deviations from intended interventions; C: Bias due to missing outcome data; D: Bias in the measurement of the outcome; E: Bias in selcetion of the reported result; F: Overall risk of bias

2) Effect of maternal nutrition supplementation versus standard care

All outcomes are included in the main text

3) Effect of mental health intervention versus standard care

All outcomes are included in the main text

4) Effect of relaxation therapy versus standard care

All outcomes are included in the main text

5) Effect of cash transfer versus standard care

All outcomes are included in the main text
